# Association between mortality and replacement solution bicarbonate concentration in continuous renal replacement therapy: A propensity-matched cohort study

**DOI:** 10.1371/journal.pone.0185064

**Published:** 2017-09-28

**Authors:** Kianoush Kashani, Charat Thongprayoon, Wisit Cheungpasitporn, Gina M. Iacovella, Abbasali Akhoundi, Robert C. Albright

**Affiliations:** 1 Division of Nephrology and Hypertension, Department of Internal Medicine, Mayo Clinic, Rochester, Minnesota, United States of America; 2 Division of Pulmonary and Critical Care, Department of Internal Medicine, Mayo Clinic, Rochester, Minnesota, United States of America; University of Sao Paulo Medical School, BRAZIL

## Abstract

**Background:**

Given the known deleterious effects seen with bicarbonate supplementation for acidemia, we hypothesized that utilizing high bicarbonate concentration replacement solution in continuous venovenous hemofiltration (CVVH) would be independently associated with higher mortality.

**Methods:**

In a propensity score-matched historical cohort study conducted at a single tertiary care center from December 9, 2006, through December 31, 2009, a total of 287consecutive adult critically ill patients with Stage III acute kidney injury (AKI) requiring CVVH were enrolled. We excluded patients on maintenance dialysis, those who received other modalities of continuous renal replacement therapies, and patients that received a mixed of 22 and 32 mEq/L bicarbonate solution pre- and post-filter. The primary outcome was in-hospital and 90-day mortality rates.

**Results:**

Among enrollees, 68 were used 32 mEq/L bicarbonate solution, and 219 received 22mEq/L bicarbonate solution for CVVH. Patients on 32 mEq/L bicarbonate solution were more often non-surgical, had lower pH and bicarbonate level but had higher blood potassium and phosphorus levels in comparison with those on 22 mEq/L bicarbonate solution. After adjustment for the baseline characteristics, the use of 32 bicarbonate solution was significantly associated with increased in-hospital (HR = 1.94; 95% CI 1.02–3.79) and 90-day mortality (HR = 1.50; 95% CI 1.03–2.14). There was a significant increase in the hospital (p = .03) and 90-day (p = .04) mortality between the 22 vs. 32 mEq/L bicarbonate solution groups following propensity matching.

**Conclusion:**

Our data showed there is a strong association between using high bicarbonate solution and mortality independent of severity of illness and comorbid conditions. These findings need to be evaluated further in prospective studies.

## Introduction

Acute kidney injury (AKI) is a very common complication among intensive care unit (ICU) patients, and it is associated with a significant mortality and morbidity. [1, 2] The severity and length of AKI are directly related to the patient death. [3] Patients who require renal replacement therapy (RRT) for AKI have a higher mortality in comparison with those without a need for RRT. Approximately, 295 cases per million/year develop AKI require RRT. [1, 4] In a recent study, 18% of patients who developed AKI stage III (based on serum creatinine and urine output criteria) developed end-stage kidney disease, and 65% of them died within one year. [3] In the recent years, there have been several studies to compare different modalities of RRT, [5–10] the timing of RRT initiation, [11, 12] and the appropriate dose of RRT. [13, 14] Despite significant progress in the field, there are many questions regarding the optimum modality of RRT (i.e. intermittent hemodialysis versus continuous renal replacement therapy [CRRT]) and its prescription characteristics.

In a recent report, we described the incidence of adverse events that may be associated with CRRT. [15] We reported the rates of ICU and hospital mortality as 40% and 51%, respectively. Although, the high rate of death in this cohort is mainly due to the severity of critical illness (APACHE III; median Interquartile Range [IQR] in this cohort was 109; IQR, 91–130) and AKI; we seek modifiable factors, which can affect patient outcomes including CRRT prescription characteristics. One such factor of the CRRT prescription is the bicarbonate concentration in the replacement solutions. The higher concentration of bicarbonate is used when patients suffer from more severe acidemia or its associated symptoms. Severe acidemia is defined as arterial plasma with a pH of less than 7.2. [16, 17] The rapid development of severe acidemia could cause cerebral edema, respiratory failure, cardiac dysfunction, systemic vasoplegia, and pulmonary vasoconstriction. [17–20] To avoid such effects of rapid, severe acidemia, alkalinizing agents, mainly bicarbonate, are frequently utilized to normalize the systemic pH, quickly. Conversely, the use of bicarbonate has been associated with a higher incidence of sodium and fluid overload, an increase in lactate and carbon dioxide production and decreases in both serum ionized calcium and seizure threshold. Arguments are additionally made with respect to a deleterious paradoxical central nervous system acidosis when bicarbonate is supplemented for lactic acidosis. Ultimately, firm data regarding the beneficial association of bicarbonate utilization on the outcomes of CRRT patients is lacking. In the surviving sepsis campaign, authors recommend “Not using sodium bicarbonate therapy for the purpose of improving hemodynamics or reducing vasopressor requirements in patients with hypoperfusion-induced lactic acidemia with pH ≥7.15.” [16]

During critical illnesses, reactive oxygen species (ROS) participate in worsening organ failure and the patients’ outcomes. Some authors suggest bicarbonate can act as a pro-oxidant agent, which could potentially enhance the adverse effect of ROS during critical illness. [21]

For the ICU patients with AKI, who require RRT and are hemodynamically unstable, CRRT is recommended as the modality of choice. [22] While patients are on CRRT, they receive large quantities of bicarbonate from the replacement fluids or dialysate. Clinicians can choose different concentrations of bicarbonate in such fluids to accommodate the patients’ needs and provide them a more suitable metabolic milieu for recovery. In this study, we report the results of a retrospective propensity matched cohort study of patients who underwent CRRT during an ICU admission to evaluate the impact of the bicarbonate concentration in the replacement fluid on the patients’ mortality and other ICU outcomes.

## Methods

### Study population and setting

We included all consecutive adult patients who were admitted to the ICUs at Mayo Clinic Hospital—Rochester and underwent CRRT from December 9, 2006, through December 31, 2009. Only the first CRRT treatment of each unique patient was included in the analysis. We excluded those who received a combination of 22mEq/L and 32mEq/L bicarbonate replacement solutions during the CRRT treatment, were on intermittent hemodialysis or peritoneal dialysis prior to hospitalization and received either continuous venovenous hemodialysis (CVVHD) or continuous venovenous hemodiafiltration (CVVHDF). Also, patients with no research authorization, pregnant women, and prisoners were excluded. The study was reviewed and approved by the Mayo Clinic Institutional Review Board, and informed consent was waived.

### Data collection

Clinical characteristics, demographic information, and laboratory data were collected using manual and automated retrieval of institutional electronic health records. These included age, sex, race, body mass index (BMI), chronic comorbidities, ICU admission type, AKI etiology, the use of vasopressor and mechanical ventilator at RRT start, and the most recent laboratory data within 24 hours prior to the RRT start. The Charlson Comorbidity Score was computed to assess comorbidities at the time of the RRT start. The primary outcome was all-cause mortality within 90 days after the RRT start. Other outcome variables included ICU mortality, in-hospital mortality, ICU and hospital length of stay, and CRRT duration.

### CRRT characteristics

Continuous venovenous hemofiltration was utilized for all patients who require CRRT unless clinicians decide otherwise. Standard CRRT orders in our institution include a blood flow rate (Qb) of 200mL/min and replacement fluid rate of 30 mL/kg/hour, of which 50% is delivered as pre-dialyzer and 50% as post-dialyzer. We used two different calcium-free replacement solutions with 22mEq/L and 32 mEq/L of bicarbonate concentration (PrismaSATE, BGK 4/0/1.2 and PrismaSATE, B22GK 4/0; Gambro^®^). We utilized regional citrate anticoagulation with a dextrose citrate solution formula A (ADC-A) to achieve 3mmol of citrate for each liter of blood flow with a dose adjustment in patients with severe liver failure.

### Statistical analysis

All continuous variables were summarized as the mean and standard deviation (SD). All categorical or ordinal data were summarized as count and percentages. The differences in clinical characteristics, laboratory data prior to RRT initiation, and outcomes between patients treated with a 22mEq/L and 32mEq/L bicarbonate solution were assessed using unpaired t-test for continuous variables and Chi-squared test for categorical variables. The patient survival, from the initiation of RRT to death or the last follow-up time (up to 90-days after RRT initiation), was presented using the Kaplan-Meier plot and compared using the log-rank test. The association between bicarbonate solution group used and the mortality was assessed using a multivariate Cox proportional hazard analysis. The hazard ratio (HR) with 95% confidence interval (CI) is reported. The HR was adjusted for variables with statistically significant differences between the groups in a univariate analysis. The adjusting variables were the ICU admission type, pH, bicarbonate, potassium, and phosphorus. A two-sided *P* value of <.05 was considered statistically significant. Unless specified, the analyses were performed using JMP statistical software (version 10, SAS, Cary, NC).

**Fig 1:**
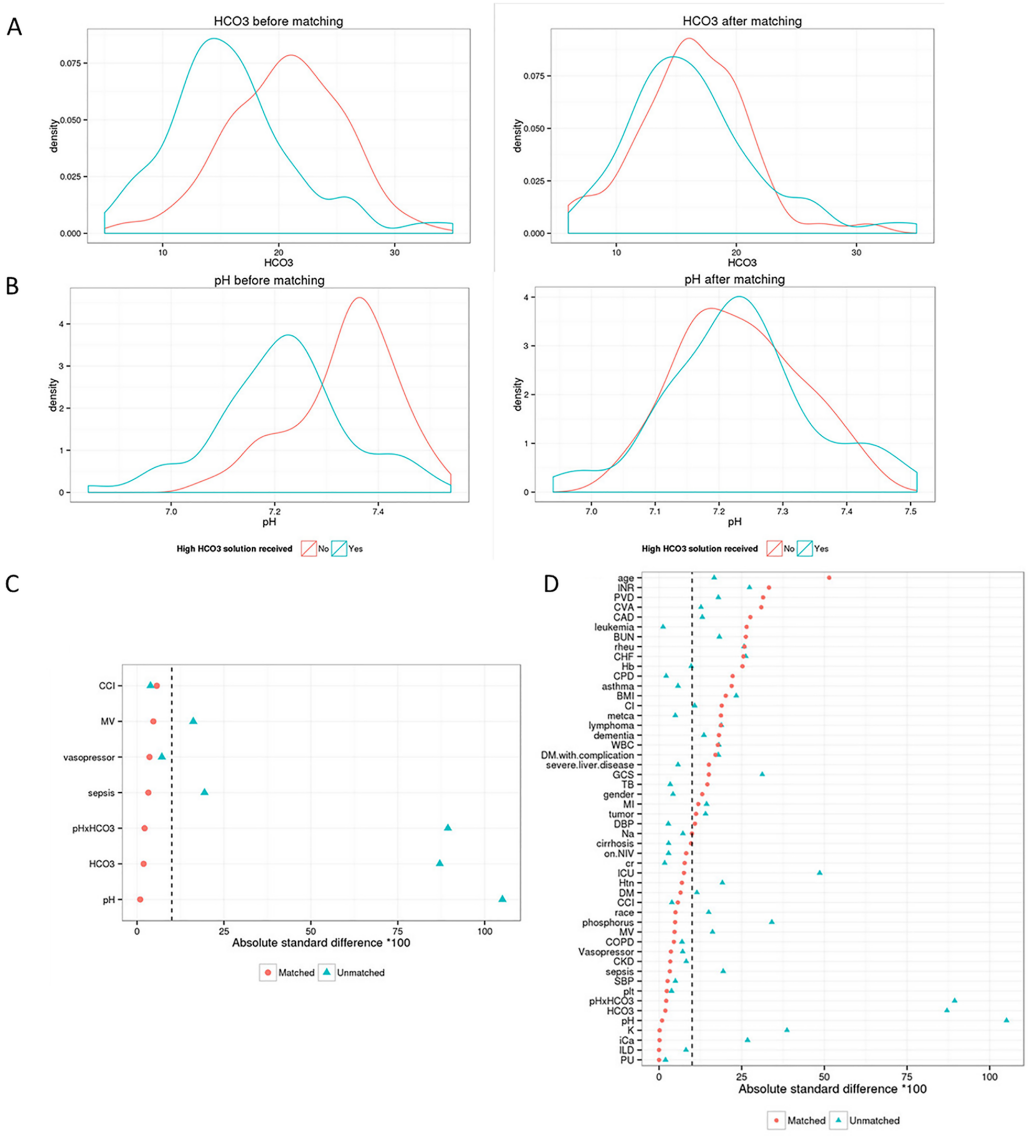
Distribution of A) Bicarbonate and B) pH before and after propensity matching. C) Absolute standardized difference plot for variables identified as most important for matching. D) Absolute standardized difference scheme for all variables in the dataset. Abbreviations: CCI, Charlson Comorbidity Index; MV, Mechanical Ventilation; INR, International Normalized Ratio; PVD, Peripheral Vascular Disease; CVA, Cerebrovascular Accident; CAD, Coronary Artery Disease; BUN, Blood Urea Nitrogen; Rheu, History of Rheumatologic Diseases; CHF, Congestive Heart Failure; Hb, Hemoglobin; CPD, Chronic Pulmonary Disease; BMI, Body Mass Index; Cl, Chloride; metca, Metastatic Cancer; WBC, White Blood Cell; DM, Diabetes Mellitus; GCS, Glasgow Coma Scale; TB, Total Bilirubin; MI, Myocardial Infarction; DBP, Diastolic Blood Pressure; NIV, Non-Invasive Ventilation; ICU, Intensive Care Unit; Htn, Hypertension; COPD, Chronic Obstructive Lung Disease; CKD, Chronic Kidney Disease; SBP, Systolic Blood Pressure; ILD, Interstitial Lung Disease; PU, Peptic Ulcer.

To further mitigate selection biases and potential confounders between 22mEq/L and 32mEq/L bicarbonate solution groups, we compared the two groups after propensity score matching (62 matched pairs). [23] The initial cohort prior to matching contained 287 patients (219 patients in the 22mEq/L bicarbonate solution group, and 68 patients in the 32mEq/L bicarbonate solution group). Propensity scores were estimated using logistic regression by using pH, bicarbonate, an interaction term between pH and bicarbonate, the presence of sepsis, use of mechanical ventilation, vasopressor administration, intra-aortic balloon pump (IABP), and Charlson Comorbidity Index as covariates. Matching was performed in R software (version 3.1.1; Vienna, Austria) using the Matching package. [24] Patients in 22mEq/L and 32mEq/L bicarbonate solution groups were matched on a 1:1 basis on the logit of the propensity score with a caliper width equal to 0.2 of the SD of the logit of the propensity score. [25] Covariate balance in the matched sample was assessed using the absolute standard difference (ASD), with an ASD of less than 0.1 considered to denote a negligible imbalance in covariates between the treatment groups. [26, 27] Fig 1 describes the baseline bicarbonate and pH, along with the absolute standardize differences before and after propensity matching.

## Results

Among the 595 patients who received CRRT during the study period, 287 patients who received either 22mEq/L or 32mEq/L bicarbonate solution entered the final analysis. In this cohort, 177 (62%) were male, and 237 (83%) were Caucasian. A limited deidentified dataset is provided in S1 Table. The mean (±SD) age, BMI, and Charlson Comorbidity score among this cohort was 62±14 years, 32.6±9.4kg/m^2^, and 3.4±2.6; respectively. Bicarbonate concentration of 22mEq/L was prescribed in 219 patients while 68 received only the 32mEq/L bicarbonate solution. Patients who received the 32mEq/L bicarbonate solution in comparison with those who received the 22mEq/L bicarbonate solution were more often admitted to the medical ICU (78% vs. 56% for 22mEq/L bicarbonate, *P* = .001), had a lower pH (7.22 vs 7.34, *P*<.001), and had a lower bicarbonate level (16 vs. 21mEq/L, *P*<.001), and had higher potassium and phosphorus levels. Table 1 summarizes the baseline characteristics of the final cohort prior to and after the propensity matching. S1 Fig shows the changes in acid-base related laboratory data during the first 7 days of CRRT in the matched and full cohorts. S2 Fig shows changes in pH among patients who had metabolic alkalosis during the CRRT treatment. S3 Fig indicates the fluid balance and CRRT dose during the first 7 days of CRRT treatment.

**Table 1:** Baseline characteristics in full and matched cohort.

Baseline Characteristics	Full cohort			Matched cohort		
	Bicarbonate		*P* value	Bicarbonate		*P* value
	32mEq/L (n = 68)	22mEq/L (n = 219)		32mEq/L (n = 62)	22mEq/L (n = 62)	
Age, year, mean(±SD)	64 (±14)	62 (±14)	.23	64 (±14)	62 (±14)	.30
Male sex, n (%)	43 (63)	134 (61)	.76	37 (60)	33 (53)	.47
White, n (%)	59 (87)	178 (81)	.30	54 (87)	55 (89)	.78
BMI, kg/m^2^, mean(±SD)	31 (±9)	33 (±10)	.09	31 (±9)	33 (±11)	.26
Comorbidity, n (%)							
Diabetes Mellitus	28 (41)	78 (36)	.41	27 (43)	25 (40)	.72
Hypertension	46 (68)	128 (58)	.18	43 (69)	41 (66)	.70
CAD	18 (26)	71 (32)	.35	17 (27)	10 (16)	.13
CHF	9 (13)	51 (23)	.08	8 (13)	14 (23)	.16
Cardiovascular Disease	10 (15)	23 (11)	.34	10 (16)	4 (6)	.15
PVD	7 (10)	12 (5)	.16	7 (11)	2 (3)	.16
Chronic Liver Disease	14 (21)	47 (21)	.88	13 (21)	19 (31)	.22
Cirrhosis	10 (15)	30 (14)	.83	9 (15)	7 (11)	.58
Charlson score, mean (±SD)	3.3 (±2.5)	3.4 (±2.7)	.78	3.5 (±2.5)	3.7 (±3.1)	.75
Medical ICU, n (%)	53 (78)	122 (56)	.001	48 (77)	46 (74)	.67
Vasopressor, n (%)	50 (74)	154 (70)	.61	45 (73)	44 (71)	.84
Bicarbonate bolus, n (%)	20 (30)	112 (51)	.002	4 (6)	24 (38)	.2
Bicarbonate infusion, n (%)	12 (18)	11 (5)	.002	7 (11)	17 (27)	.5
Dose of bicarbonate infusion, mEq/day, median (IQR)	283 (104–4276)	265 (165–1277)	.4	330 (85–2688)	211 (151–1140)	.4
Mechanical ventilator, n (%)	59 (87)	177 (81)	.26	53 (85)	54 (87)	.79
Septic AKI, n (%)	37 (54)	98 (45)	.16	35 (56)	36 (58)	.86
Laboratory findings, mean (±SD)						
pH	7.22(±0.13)	7.34(±0.10)	<.001	7.23(±0.12)	7.23(±0.09)	.96
HCO3, mEq/L	16 (±6)	21 (±5)	<.001	16 (±6)	16 (±5)	.92
BUN, mg/dL	56 (±33)	62 (±33)	.19	57 (±34)	66 (±34)	.15
Creatinine, mg/dL	3.1 (±1.4)	3.1 (±1.4)	.90	3.2 (±1.5)	3.3 (±1.3)	.67
Sodium, mmol/L	139 (±7)	140 (±7)	.61	139 (±7)	138 (±6)	.58
Potassium, mmol/L	4.8 (±1.1)	4.5 (±0.8)	.01	4.8 (±1.1)	4.8 (±0.8)	.99
Chloride, mmol/L	106 (±5)	106 (±6)	.67	106 (±5)	105 (±6)	.75
Lactate, mmol/L	5.6 (±6)	4 (±4)	.052	4.8 (±4.7)	4.2 (±4.5)	.52
Ionized Calcium, mg/dL	4.4 (±0.8)	4.6 (±0.8)	.06	4.4 (±0.8)	4.4 (±0.5)	.99
Phosphorus, mg/dL	6.3 (±2.8)	5.4 (±2.5)	.02	6.1 (±2.6)	6.3 (±3.5)	.79
Hemoglobin, g/dL	9.9 (±1.8)	10.0 (±1.7)	.49	9.8 (±1.7)	10.3 (±1.9)	.16
Platelet, /L	131 (±100)	128 (±94)	.79	123 (±94)	126 (±95)	.90
INR	1.9 (±1.1)	1.7(±0.7)	.08	2.0 (±1.1)	1.7 (±0.7)	.07
Total Bilirubin, mg/dL	4.4 (±7.9)	4.2 (±6.8)	.82	4.5 (±8.2)	3.5 (±6.1)	.42

Abbreviations: BUN, Blood Urea Nitrogen; CAD, Coronary Artery Disease; CHF, Congestive Heart Failure; ICU, Intensive Care Unit; INR, International Normalized Ratio; PVD, Peripheral Vascular Disease; SD, Standard Deviation

Prior to the propensity matching, the in-hospital and 90-day mortalities were significantly higher in those who received the 32mEq/L bicarbonate solution when it was compared with those who received the 22mEq/L bicarbonate solution (*P* = .01), see Table 2. After adjusting for the ICU admission type, pH, bicarbonate, potassium, and phosphorus, the use of the 32mEq/L bicarbonate solution remained significantly associated with increased in-hospital mortality (HR, 1.94; 95% CI, 1.02–3.79; *P* = .04) and 90-day mortality (HR = 1.50; 95% CI, 1.03–2.14; *P* = .03). Among patients who survived the hospital (n = 127), 39 (31%) patients remained on RRT.

**Table 2:** Outcomes between 32 and 22 mEq/L bicarbonate groups in full and matched cohort.

Outcomes	Full cohort			Matched cohort		
	Bicarbonate		*P* value	Bicarbonate		*P* value
	32mEq/L (n = 68)	22 mEq/L (n = 219)		32 mEq/L (n = 62)	22 mEq/L (n = 62)	
ICU mortality, n (%)	36 (53)	90 (41)	.09	32 (52)	30 (48)	.72
In-hospital mortality, n (%)	47 (69)	113 (52)	.01	42 (68)	30 (48)	.03
90-day mortality, n (%)	52 (76)	128 (58)	.01	47 (76)	37 (60)	.04
ICU stay (day), mean (±SD)	10 (±1)	13 (±1)	.04	9 (±9)	12 (±12)	.2
Hospital stay, n of days, mean (±SD)	26 (±4)	30 (±2)	.4	26 (±35)	26 (±26)	.96
CRRT duration, n of days, mean (±SD)	5 (±0.8)	6 (±0.4)	.2	5 (±6)	5 (±4)	.8
Mechanical ventilator, n of days, mean (±SD)	8 (±1.2)	10 (±0.7)	.1	7 (±1)	9 (±1)	.3

Abbreviations: CRRT, Continuous Renal Replacement Therapy; HCO3, Bicarbonate; ICU, Intensive Care Unit; SD, Standard Deviation

Among the 62 propensity-matched pairs, all baseline characteristics prior to the RRT initiation in patients treated with both bicarbonate solutions were comparable, including the blood bicarbonate level (16±6 vs. 16±5; *P* = .92) and the pH level (7.23±0.12 vs. 7.23±0.09; *P* = .96), see Table 1.

**Fig 2:**
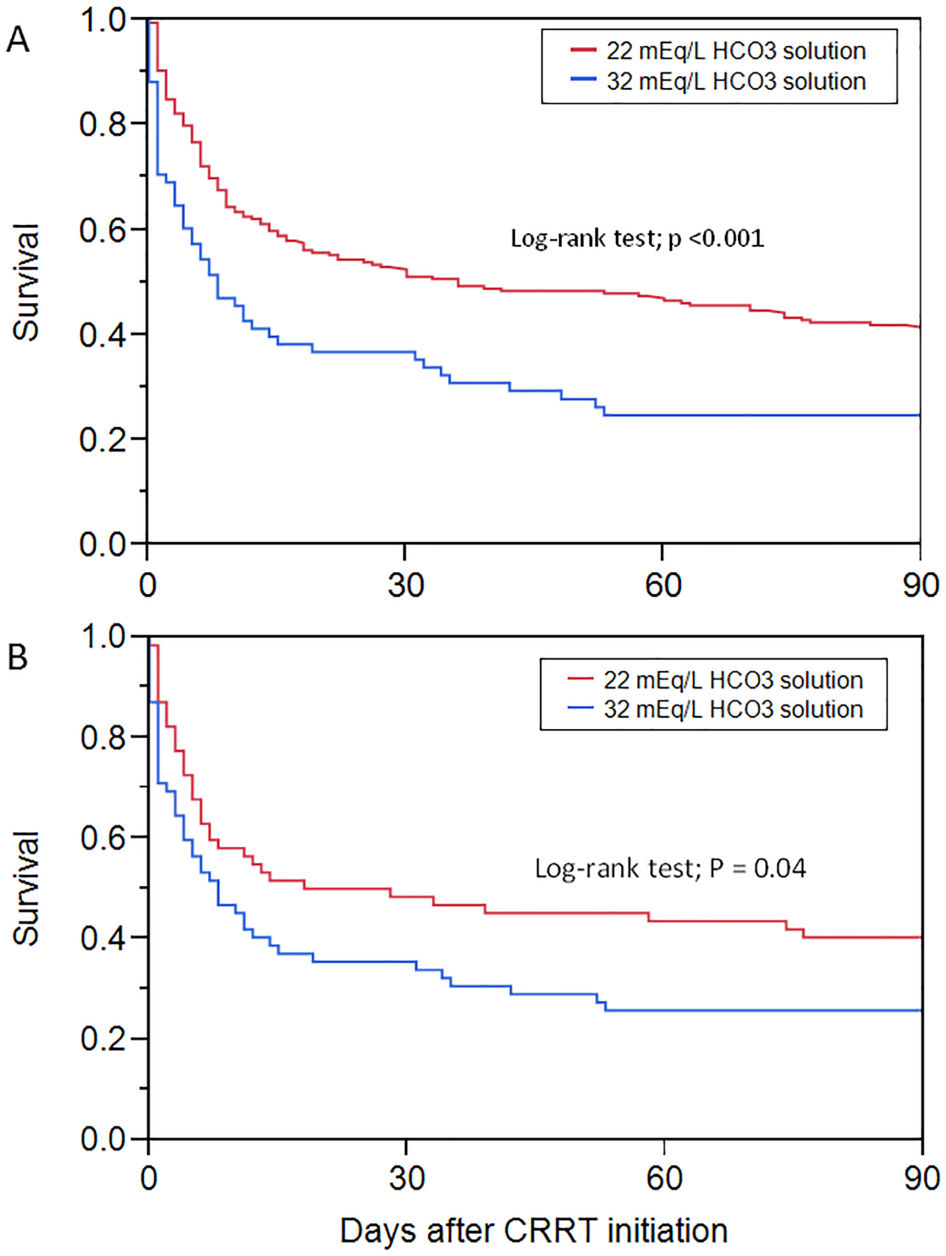
Kaplan-Meier plot is demonstrating survival in 32mEq/L vs. 22mEq/L HCO3 solution groups. A) Full cohort adjusted for ICU type, pH, HCO3, K, and phosphorus. The Hazard Ratio is 1.76 (95% CI, 1.26–2.42; *P*<.001) and the Adjusted Hazard Ratio* was 1.50 (95% CI, 1.03–2.14; *P* = .03). B) Propensity-matched cohort with a Hazard Ratio of 1.55 (95% CI, 1.01–2.41; *P* = .04). *adjusted for ICU type, pH, HCO3, K, and phosphorus. Abbreviations: HCO3, Bicarbonate; ICU, Intensive Care Unit; K, Potassium.

In the matched cohort, both in-hospital and 90-day mortalities remained significantly higher in patients who received the 32mEq/L bicarbonate solution (*P* = .03 and .04, respectively), shown in Table 2. Although the ICU length of stay was significantly longer among those who received the 22mEq/L bicarbonate solution (*P* = .04) before propensity matching, there was no statistically significant difference after propensity matching. Also, the ICU mortality, hospital length of stay, days on CRRT and the duration of the mechanical ventilator were not different between the two cohorts before or after propensity matching. Kaplan-Meier plots in the full cohort and the matched cohort indicate significant differences in the 90-day mortality among the two groups (Fig 2).

## Discussion

In this study, we report the effect of the bicarbonate concentration in the replacement solution on the ICU, hospital, and 90-day mortality rates in a large cohort of ICU patients who required CRRT for AKI. We found the high bicarbonate concentration in the replacement solution is an independent risk factor for the hospital and 90-day mortalities. The difference in mortality remained statistically significant following a successful propensity score matching between the low and high bicarbonate concentration cohorts. There were no differences in the ICU, hospital length of stay, the need for and duration of mechanical ventilation, and the number of days on the CRRT.

Continuous RRT has become a standard-of-care in many institutions for treatment of AKI among critically ill patients in the ICU, particularly those who are hemodynamically unstable or suffer from intracranial hypertension. Despite the widespread utilization of CRRT, there are essential questions regarding the optimized time of initiation, mode and dose of CRRT, and other prescription characteristics that remain unanswered. In this study, we described the role of replacement fluid bicarbonate concentration on patient outcomes. Although data is available for the intravenous prescription of bicarbonate (its benefits and side effects), the data on the delivery of bicarbonate via RRT is scarce. In a study by Rocktäschel et al., the authors concluded that continuous venovenous hemofiltration (CVVH) can correct metabolic acidosis within 24 hours, mainly by the removal of unmeasured anions including phosphate and chloride. Interestingly, the authors noted many patients on CVVH developed metabolic alkalosis within 72 hours. [28]

Our findings are consistent with previous discoveries regarding the potential harmful effects of a bicarbonate prescription in the critically-ill patients. [29] Although severe acidemia is associated with significant complications, such as decreased mean arterial pressure, impaired coronary artery perfusion and myocardial contractility, [30] and higher mortality, the rapid delivery of bicarbonate in order to correct acidemia could be linked to clinically relevant side effects. In small randomized double blinded studies, bicarbonate infusion was not able to improve the hemodynamic state of critically-ill patients. [31, 32] Furthermore, a bicarbonate prescription to manage organic metabolic acidosis (e.g. lactic acidosis) could result in further increase in blood lactate level. One hypothesis to describe this observation is the shift of lactate from the intracellular space to the extracellular compartment. [33] Also, rapid infusion of bicarbonate would lead to a biphasic response in blood pressure. Due to the direct effect of hyperosmolarity induced by bicarbonate and a reflex-mediated mechanism blood pressure reaches a peak around 22±1.5 seconds and then returns to the baseline by 120 seconds. [34] Also, the bicarbonate infusion could attenuate the effect of vasoactive agents, e.g. epinephrine. [35] In other reports, sodium bicarbonate administration found to be associated with angina or ST-segment elevation due to increased myocardial oxygen consumption, promoting demand/perfusion mismatching. [36, 37] Administration of bicarbonate leads to increased carbon dioxide production. Indeed, 100mEq of bicarbonate produces about 2.2 liters of carbon dioxide, which is tenfold in comparison to its production in a healthy individual per minute. [38] As a result, the paradoxical cerebral acidosis or uncoupling of the arterial and cerebrospinal fluid (CSF), pH, and carbon dioxide is considered a deleterious effect of rapid administration of bicarbonate. [39] In animal studies, intracellular hypercarbia has been found to be associated with an attenuated myocardial contractility. [40, 41] Bicarbonate infusion is found to be linked with intracranial hypertension, which starts in about 20 seconds following administration and persists up to 20 minutes[42] and it also leads to hypernatremia, hypokalemia, and hyperosmolarity.[29] Although there is no specific published evidence demonstrating causality between higher bicarbonate dose in CRRT with higher mortality, we extrapolated the data from bicarbonate infusion to hypothesize that higher bicarbonate delivery during CRRT could be potentially associated with harmful effects which resulted in the higher observed mortality among the group who received high bicarbonate doses while on CRRT. The observed 90-day outcomes are potentially a reflection of this putative deleterious hemodynamic physiology and oxidative stress which resulted in subtle changes in recovery of kidney injury or other organ functions in our cohort.

Our study is the subject of several limitations. The most important limitation of the study is the retrospective nature of the design. Clinicians were free to choose one bicarbonate prescription over another, and hence, one might suppose the patients who received the higher bicarbonate replacement solutions were potentially more desperately ill in the eyes of the physician teams making these choices. However, via utilization of propensity matching, we attempted to mitigate the biases associated with retrospective studies and the illness severity issue. We did not account for the amount of extra-dialysis bicarbonate administration by the primary teams, which could potentially result in bias. The decision to not include extra-dialysis bicarbonate in the analysis is mainly due to the inconsistent documentation of such prescription particularly in the cardiopulmonary resuscitation (CPR) or near-CPR conditions.

Our report also has several strengths. The vigorous propensity matching allows us to be able to compare the two closely matched groups for the effect of different bicarbonate concentrations on the outcome. Our paper is also the first report of such association in the literature, which could be used as a hypothesis-generating study for future prospective investigations.

## Conclusions

We reported a novel finding regarding the impact of a high bicarbonate solution on the mortality of critically ill patients receiving CRRT. Our results are aligned with previous reports indicating the deleterious effect of intravenous prescription of bicarbonate. Our findings could be utilized as a hypothesis-generating study for the future prospective randomized trials to delineate the side effects of a high bicarbonate solution for CRRT.

## Supporting information

S1 Table Limited deidentified dataset. (XLSX)

S1 Fig Changes of acid-base related laboratory data during the first 7 days of CRRT in the matched and full cohorts. (DOCX)

S2 Fig Changes in pH among patients who had metabolic alkalosis during the CRRT treatment. In the matched cohort, among 124 patients in the propensity-matched cohort, only 3 had metabolic alkalosis (defined as pH > 7.43). The trend of pH among these three patients is included in figure (1). Within the full cohort, among 287 patients in the propensity-matched cohort, only 8 had metabolic alkalosis (defined as pH > 7.43). The trend of pH among these three patients is included in (2).
(DOCX)

S3 Fig Fluid balance and CRRT dose during the first 7 days of treatment in the matched and full cohorts. In the matched cohort median (IQR) of serum BUN and Creatinine levels were 47 (35–70), and 2.7 (2.1–3.6) mg/dL, respectively (a). In the full cohort median (IQR) of serum BUN and Creatinine levels were 43 (28–68), and 2.5 (1.7–3.5) mg/dL, respectively (b).
(DOCX)
