# Supplementary material for: Association between mortality and replacement solution bicarbonate concentration in continuous renal replacement therapy: A propensity-matched cohort study
Source: PLoS One. 2017 Sep 28;12(9):e0185064. doi: 10.1371/journal.pone.0185064 (PMC5619733; doi:10.1371/journal.pone.0185064)

Supplementary material:

S1 Fig- Changes of acid-base related laboratory data during the first 7 days of CRRT in the matched and full cohorts.

- 1. Matched cohort: please see the following figures.


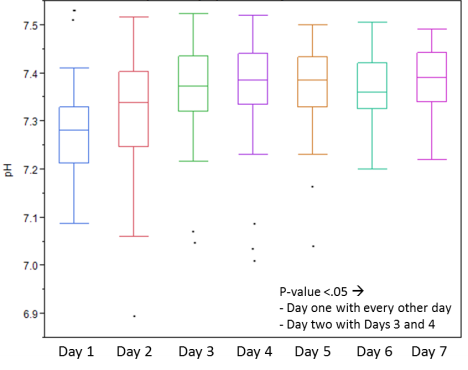

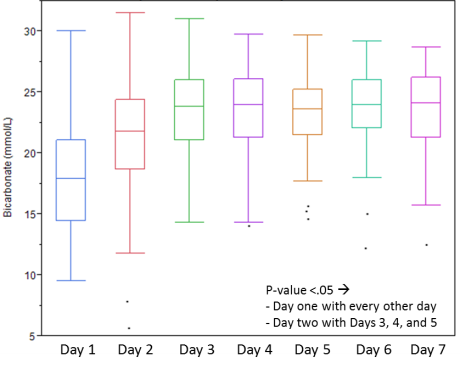


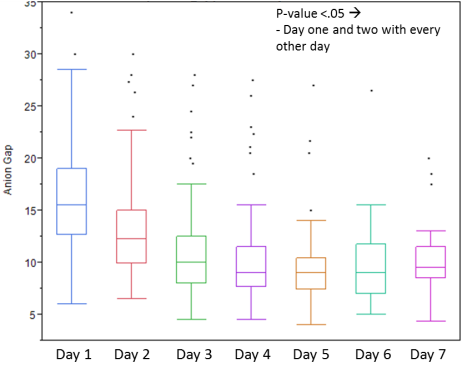

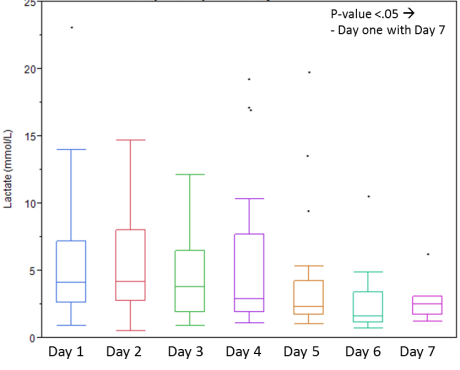


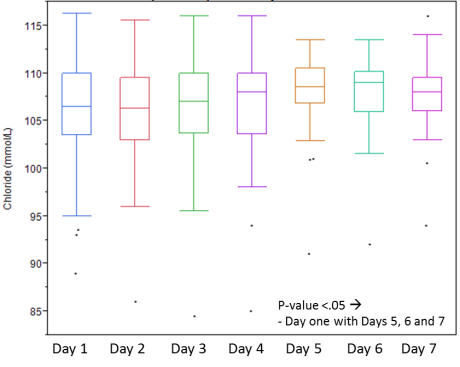

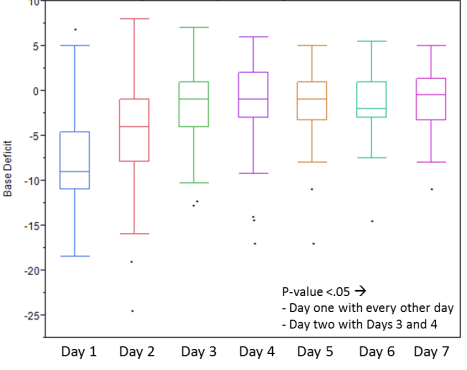


- 1. Full cohort: please see the following figures.


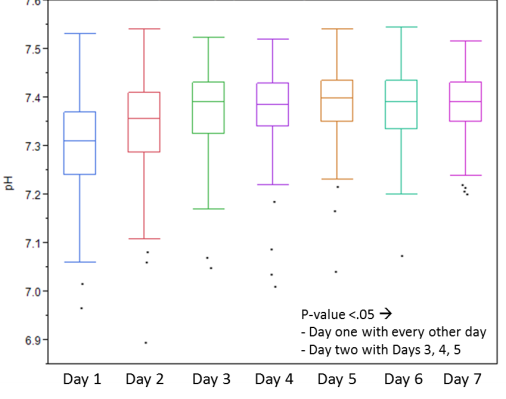

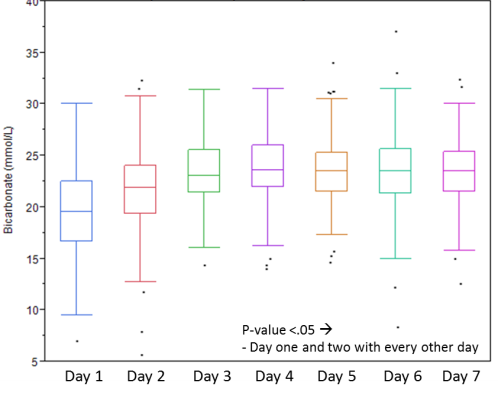


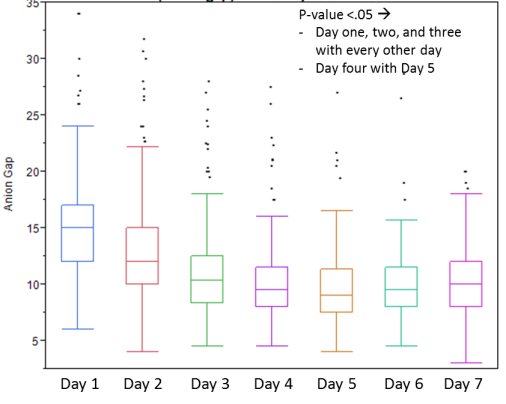

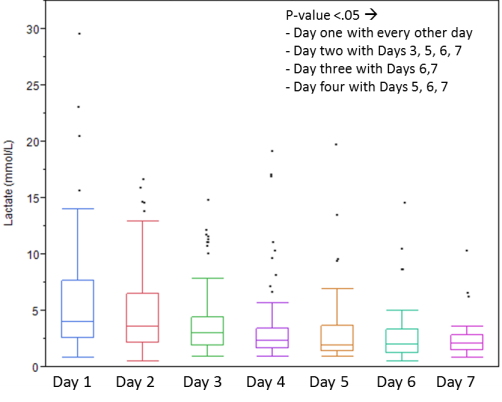


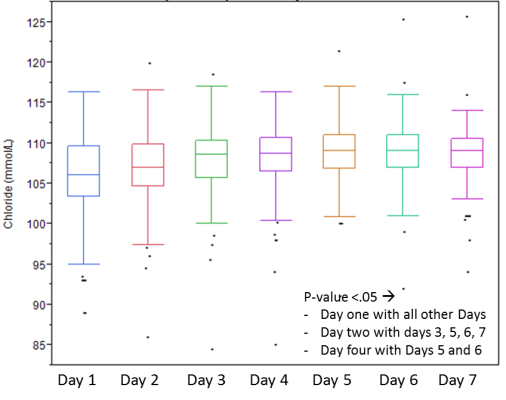

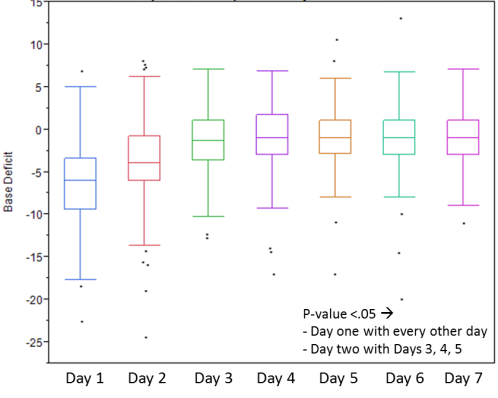

Supplement: S1 Fig — (DOCX) [file pone.0185064.s002.docx]
