# Supplementary material for: Association between mortality and replacement solution bicarbonate concentration in continuous renal replacement therapy: A propensity-matched cohort study
Source: PLoS One. 2017 Sep 28;12(9):e0185064. doi: 10.1371/journal.pone.0185064 (PMC5619733; doi:10.1371/journal.pone.0185064)

**Supplementary material:**

S2 Fig: Changes in pH among patients who had metabolic alkalosis during the CRRT treatment. In the matched cohort, among 124 patients in the propensity-matched cohort, only 3 had metabolic alkalosis (defined as pH > 7.43). The trend of pH among these three patients is included in figure (1). Within the full cohort, among 287 patients in the propensity-matched cohort, only 8 had metabolic alkalosis (defined as pH > 7.43). The trend of pH among these three patients is included in (2).

- - 1. Only days 1 and 7 had statistical significance in their pH


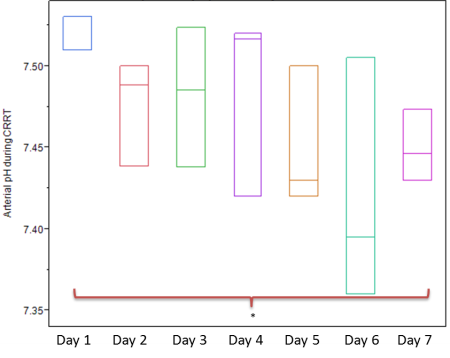


- - 1. Only days 1 and 7 had statistical significance in their pH


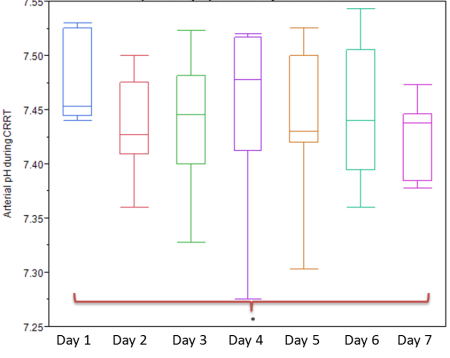

Supplement: S2 Fig — In the matched cohort, among 124 patients in the propensity-matched cohort, only 3 had metabolic alkalosis (defined as pH > 7.43). The trend of pH among these three patients is included in figure (1). Within the full cohort, among 287 patients in the propensity-matched cohort, only 8 had metabolic alkalosis (defined as pH > 7.43). The trend of pH among these three patients is included in (2). (DOCX) [file pone.0185064.s003.docx]
