# Supplementary material for: Association between mortality and replacement solution bicarbonate concentration in continuous renal replacement therapy: A propensity-matched cohort study
Source: PLoS One. 2017 Sep 28;12(9):e0185064. doi: 10.1371/journal.pone.0185064 (PMC5619733; doi:10.1371/journal.pone.0185064)

**Supplementary Material:**

S3 Fig: Fluid balance and CRRT dose during the first 7 days of treatment in the matched and full cohorts. In the matched cohort median (IQR) of serum BUN and Creatinine levels were 47 (35-70), and 2.7 (2.1-3.6) mg/dL, respectively (a). In the full cohort median (IQR) of serum BUN and Creatinine levels were 43 (28-68), and 2.5 (1.7-3.5) mg/dL, respectively (b).

1. Matched cohort
2. Fluid balance


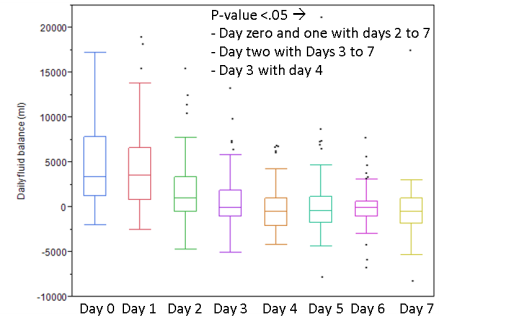


1. CRRT dose (ml/kg)


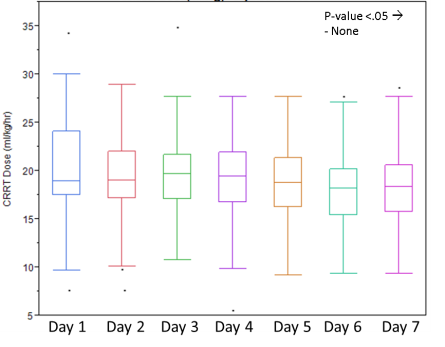


1. Full cohort
2. Fluid balance


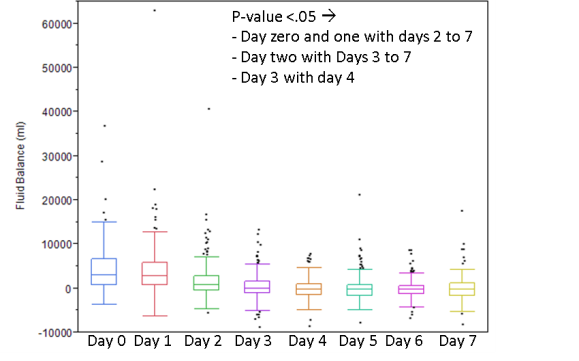


1. CRRT dose (ml/kg)


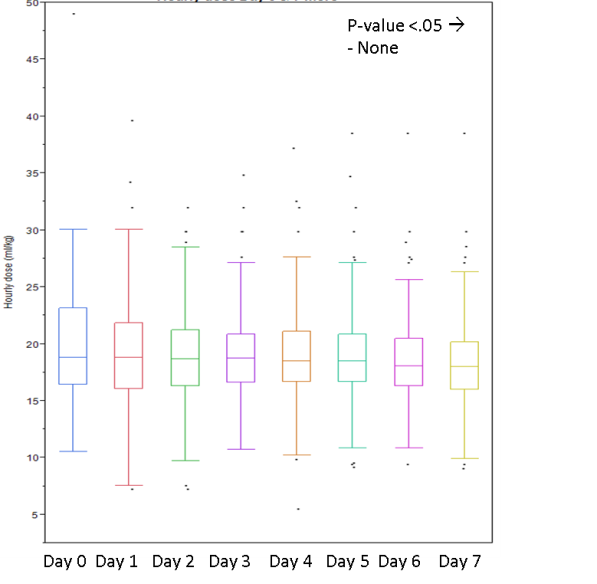

Supplement: S3 Fig — In the matched cohort median (IQR) of serum BUN and Creatinine levels were 47 (35–70), and 2.7 (2.1–3.6) mg/dL, respectively (a). In the full cohort median (IQR) of serum BUN and Creatinine levels were 43 (28–68), and 2.5 (1.7–3.5) mg/dL, respectively (b). (DOCX) [file pone.0185064.s004.docx]
